# Definition of the *Gonioctena subgeminata* species group (Coleoptera, Chrysomelidae, Chrysomelinae), with descriptions of two new species from China and Vietnam

**DOI:** 10.3897/zookeys.1032.63905

**Published:** 2021-04-16

**Authors:** Hee-Wook Cho

**Affiliations:** 1 Department of Zoology, Nakdonggang National Institute of Biological Resources, Sangju, 37242, South Korea Nakdonggang National Institute of Biological Resources Sangju Republic of Korea

**Keywords:** *
Asiphytodecta
*, leaf beetle, ovoviviparity, taxonomy

## Abstract

This paper defines and reviews the *Gonioctena subgeminata* species group of the subgenus Asiphytodecta Chen, 1935. The group contains the following five species, including two new to science: *G. subgeminata* (Chen, 1934), *G. tonkinensis* (Chen, 1934), *G. oudai* Cho & Borowiec, 2016, *G. allardi***sp. nov.**, and *G. mantillerii***sp. nov.** The species group restricted to China and Vietnam is characterized by a labrum without a tooth-like projection, elytral punctures arranged in discernible rows, and a setose aedeagus with a broad apical process. *Gonioctena tonkinensis* is newly reported as an ovoviviparous species. Habitus photographs, illustrations of diagnostic characters of each species, and a key to the species are provided.

## Introduction

The subgenus Asiphytodecta Chen, 1935, is the second most speciose group of the nine subgenera in the genus *Gonioctena*, with approximately 25 species occurring in the Oriental Region and Palearctic China (Cho and Borowiec 2016a). More than half of the *Asiphytodecta* species present a tooth-like projection at the anterior margin of the labrum and completely irregular punctures on the elytra, which are unique and remarkable characters in *Gonioctena*. There are two species groups within the subgenus, *tredecimmaculata* and *flavoplagiata*, based on the morphological characters of adults. Bezděk (2002) proposed the *G. tredecimmaculata* species group for five species occurring in the Indochinese Peninsula and China, characterized by a black median spot on the pronotum and 12 black spots on the elytra varying from small, separated spots to large, transversely merged spots. Recent taxonomic work on *Asiphytodecta* by Cho et al. (2016) and Sprecher-Uebersax and Daccordi (2016) indicated that four more species should be added to the group: *G. fraudulenta* Sprecher-Uebersax & Daccordi, *G. taiwanensis* (Achard), *G. ohmomoi* Cho, Takizawa & Borowiec, and *G. riyuetanensis* Cho, Takizawa & Borowiec. The *G. flavoplagiata* species group proposed by Cho and Borowiec (2016a) includes five species from China, Laos, and Vietnam and is easily recognized by the strongly widened and flattened last four antennomeres and two pairs of yellowish brown spots on the elytra.

In contrast, members of other *Asiphytodecta* species lacking the above unique characters have been relatively poorly studied. Moreover, an identification key for known species has not been provided, and the subgeneric placement of several species remains uncertain. This study is the first attempt to solve these taxonomic problems, and the Gonioctena (Asiphytodecta) subgeminata species group classification is proposed for five species distributed in China and Vietnam, characterized by the labrum lacking a tooth-like projection, elytral punctures arranged in discernible rows, and a setose aedeagus with a broad apical process. Two new species of the *G. subgeminata* species group were discovered by recent examination of material from the Museum National d’Histoire Naturelle, Paris and are described here.

## Materials and methods

Descriptions were prepared using Nikon SMZ800 and Nikon Eclipse E600 microscopes. Male and female genitalia were dissected from adult specimens, softened in a closed Petri dish with wet tissue paper for 12–24 hours, cleared in 10% sodium hydroxide solution, and rinsed in distilled water. Photographs were taken using the Nikon D5200 digital camera attached to the Nikon SMZ1500 microscope and were edited in Helicon Focus 5.3.12 and Adobe Photoshop CS5. Line drawings were made from photographs in Adobe Photoshop CS5 using the Wacom Intuos4 graphics tablet.

The specimens examined in this study are deposited in the following collections:

**BPBM**Bernice Pauahi Bishop Museum, Honolulu, Hawaii, USA;

**HCC** Hee-Wook Cho private collection, Yecheon, South Korea;

**HTC** Hauro Takizawa private collection, Hasuda, Japan;

**LMC** Lev N. Medvedev private collection, Moscow, Russia;

**MNHN**Museum National d’Histoire Naturelle, Paris, France;

**NHMB**Naturhistorisches Museum Basel, Basel, Switzerland;

**NHMUK**The National History Museum, London, UK;

**NMPC**Národní Muzeum, Prague, Czech Republic;

**TLMF**Horst Kippenberg private collection, Tiroler Landesmuseum Ferdinandeum, Innsbruck, Austria;

**ZMHB**Museum für Naturkunde der Humboldt-Universität, Berlin, Germany.

## Taxonomic account

### Gonioctena (Asiphytodecta) subgeminata species group

**Differential diagnosis.** Body length 5.7–8.6 mm. Body short-oval to oval, strongly convex. Ground color reddish brown to orange with black spots or sinuate transverse bands on dorsum. Last four or five antennomeres blackish brown and legs entirely reddish brown. Anterior margin of the labrum almost straight without a tooth-like projection. Elytra covered with regular rows of punctures including partially irregular ones or rather irregular punctures arranged in confused single or double rows. Aedeagus concave and setose apicolaterally, with apical process broad, produced laterally. Spermatheca C-shaped and thick, with apex widely rounded. The *Gonioctena tredecimmaculata* species group is similar to members of the *Gonioctena subgeminata* species group in body shape and coloration but differs in its completely irregular punctures on the elytra and a tooth-like projection at the anterior margin of the labrum. *Gonioctena medogana* Wang differs in the aedeagus with a very thin and glabrous apical process.

#### Key to the species of the Gonioctena (Asiphytodecta) subgeminata species group

**Table:** 

1	Elytra covered with regular punctures arranged in single rows, partially irregular (Figs 2, 3)	**2**
–	Elytra covered with irregular punctures arranged in confused single or double rows (Figs 1, 4, 5)	**3**
2	Pronotum with a pair of obscure spots (Fig. 8); apical process of aedeagus approximately 1.5 × wider than median lobe (Fig. 16). China (Sichuan)	***G. oudai* Cho & Borowiec**
–	Pronotum without obscure spots (Fig. 7); apical process of aedeagus approximately 1.2 × wider than median lobe (Fig. 15). China (Sichuan)	***G. mantillerii* sp. nov.**
3	Pronotum with a pair of basal triangular black markings (Fig. 10). China (Guangxi), Vietnam	***G. tonkinensis* (Chen)**
–	Pronotum without black markings (Figs 6, 9)	**4**
4	Elytra with 10 black spots, sometimes narrowly connected, but not merged into a band (Fig. 4). China (Anhui, Fujian, Hunan, Jiangxi, Sichuan), Taiwan	***G. subgeminata* (Chen)**
–	Elytra with two basal spots and two sinuate transverse bands (Fig. 1). Vietnam	***G. llardi* sp. nov.**

##### Gonioctena (Asiphytodecta) allardi sp. nov.

Taxon classificationAnimaliaColeopteraChrysomelidae

264E129F-1794-55DB-BF95-61F0EC699D14

http://zoobank.org/7A746AF3-58A8-4B6C-9E5A-B87E64BB98A9

**Figures 1–5.**
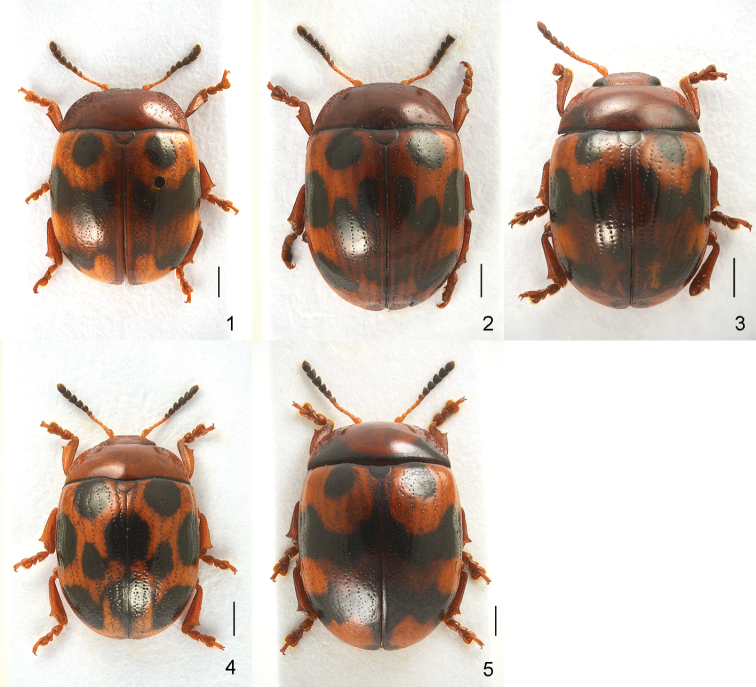
Dorsal habitus **1***Gonioctena allardi* sp. nov., holotype **2***G. mantillerii* sp. nov., holotype **3***G. oudai***4***G. subgeminata***5***G. tonkinensis*. Scale bars: 1.0 mm.

**Figures 6–10.**
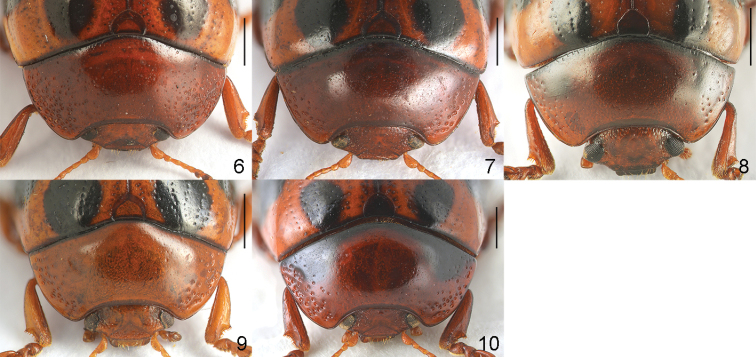
Head and pronotum **6***Gonioctena allardi* sp. nov., holotype **7***G. mantillerii* sp. nov., holotype **8***G. oudai***9***G. subgeminata***10***G. tonkinensis*. Scale bars: 1.0 mm.

**Figures 11–13.**
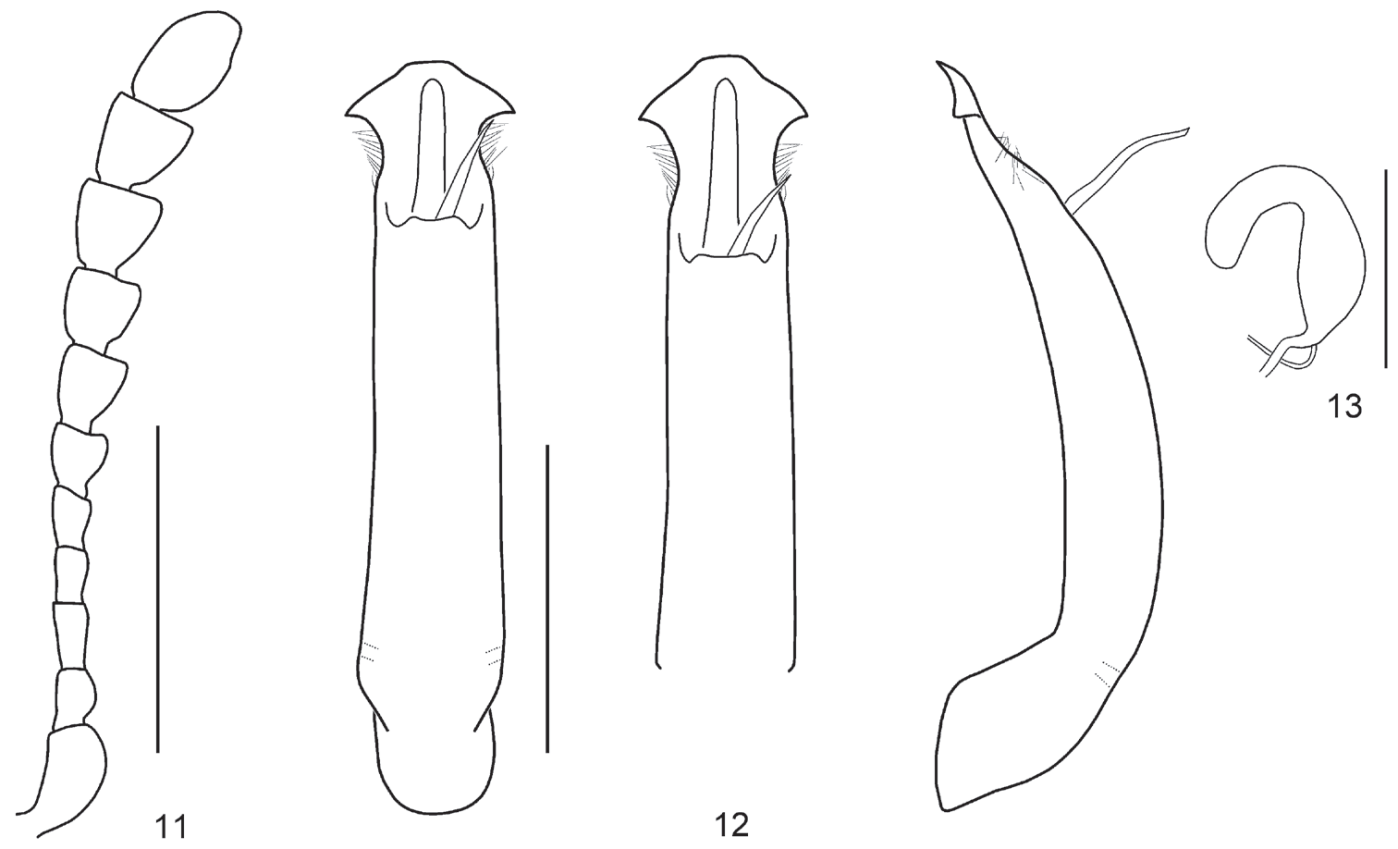
*Gonioctena allardi* sp. nov., holotype **11** antenna **12** aedeagus (dorsal view, apex in dorsal view, lateral view) **13** spermatheca. Scale bars: 1.0 mm (**11**, **12**); 0.5 mm (**13**).

Figures 1 , 6 , 11 , 12

###### Type locality.

Vietnam: Hanoi, Son Tay.

###### Type material.

***Holotype*:** ♂ (MNHN), “Son Tay // Ex. Musaeo E. Allard 1899 // MUSEUM PARIS 1952 Coll. R. Oberthur // HOLOTYPUS Gonioctena (A.) allardi sp. nov. Cho & Borowiec 2014”. ***Paratype***: 1♀ (MNHN), same data as for holotype.

###### Diagnosis.

This new species is similar to *Gonioctena mantillerii* sp. nov. and *G. oudai* in body shape and coloration. However, *G. allardi* sp. nov. can be distinguished by the following characters: elytra covered with rather irregular punctures arranged in confused single or double rows (regular punctures arranged in single rows, partially irregular in other species); pronotum without spots (same in *G. mantillerii* sp. nov., a pair of lateral obscure spots present in *G. oudai*); aedeagus with an apical process widened to lateral tooth-like projections, 1.2 × wider than the median lobe (widened to lateral blunt projections, 1.2 × wider than the median lobe in *G. mantillerii* sp. nov., very large, 1.5 × wider than the median lobe in *G. oudai*).

###### Description.

Measurements in mm (n = 2): length of body: 6.50–7.50 (mean 7.00); width of body: 5.00–5.80 (mean 5.40); height of body: 3.30–4.00 (mean 3.65); width of head: 1.95–2.10 (mean 2.03); interocular distance: 1.25–1.35 (mean 1.30); width of apex of pronotum: 2.30–2.40 (mean 2.35); width of base of pronotum: 4.42–5.00 (mean 4.71); length of pronotum along midline: 2.00–2.25 (mean 2.13); length of elytra along suture: 4.90–5.90 (mean 5.40).

Body short oval and strongly convex (Fig. 1). Head reddish brown, with labrum partially dark brown, apex of mandibles black. Antennomeres I–VI yellowish brown, VI partially darkened, VII–XI blackish brown. Pronotum reddish brown, basal margin black. Scutellum reddish brown. Elytra orange, with a pair of black spots and sinuate transverse bands, dark area between bands, tip partially darkened. Venter and legs entirely reddish brown. ***Head*.** Vertex weakly convex, covered with sparse punctures, becoming coarser and denser toward sides. Frontal suture V-shaped, reaching anterior margin, coronal suture rather short. Frons flat, suddenly depressed at anterior margin, covered with dense punctures. Clypeus very narrow and trapezoidal. Anterior margin of labrum almost straight. Mandibles with two sharp apical teeth and deep excavation for apical maxillary palpomere on outer side. Maxillary palps four-segmented, with apical palpomere slightly widened, truncated apically. Antennae reaching pronotal base; antennomere I robust; antennomere II shorter than III; antennomere III longer than IV; antennomeres VII–X widened, VIII–X each almost as long as wide; antennomere XI longest, ~ 1.51 × as long as wide (Fig. 11). ***Pronotum*.** Lateral sides widest at base, strongly and roundly narrowed anteriorly, anterior angles strongly produced (Fig. 6). Anterior and lateral margins bordered; lateral margins hardly visible in dorsal view. Trichobothria absent on both anterior and posterior angles. Disc covered with sparse punctures; lateral sides covered with much larger and denser punctures; interspaces covered with fine and sparse punctures. Scutellum distinctly wider than long, narrowed posteriorly. ***Elytra*.** Lateral sides slightly widened posteriorly, widest before middle, thence roundly narrowed posteriorly. Humeral calli well developed. Disc covered with rather irregular punctures arranged in confused single or double rows in median region, regular punctures arranged in rows in lateral region, dense punctures between second and third striae in apical half; interspaces covered with fine and sparse punctures. Epipleura visible except near base in lateral view. Hind wings well developed. ***Venter*.** Hypomera weakly rugose, with few punctures near anterolateral corners of prosternum. Prosternum covered with coarse and dense punctures bearing long setae; prosternal process enlarged apically, bordered laterally, with sparse punctures. Metasternum covered with small and sparse punctures in median region, large and dense punctures in lateral region. Abdominal ventrites covered with sparse or moderately dense punctures bearing short setae. ***Legs*.** Moderately robust. Tibiae widened apically, with a tooth-like projection. Fore legs with tarsomere I slightly narrower than III in male and distinctly narrower than III in female. Tarsal claws appendiculate. ***Genitalia*.** Aedeagus rather thin, subparallel-sided, weakly narrowed before apical process, setose apicolaterally, with apical process widened to lateral tooth-like projections in dorsal view; moderately curved, apex pointed in lateral view (Fig. 12). Spermatheca C-shaped, swollen basally, with apex rounded (Fig. 13).

###### Etymology.

Named after its collector, E. Allard.

###### Distribution.

Vietnam (Hanoi).

##### Gonioctena (Asiphytodecta) mantillerii sp. nov.

Taxon classificationAnimaliaColeopteraChrysomelidae

A09D2EA9-977F-5381-A57D-179842CFD333

http://zoobank.org/7BE92F7A-9B6A-4299-A95D-43A49969FDF1

**Figures 14, 15.**
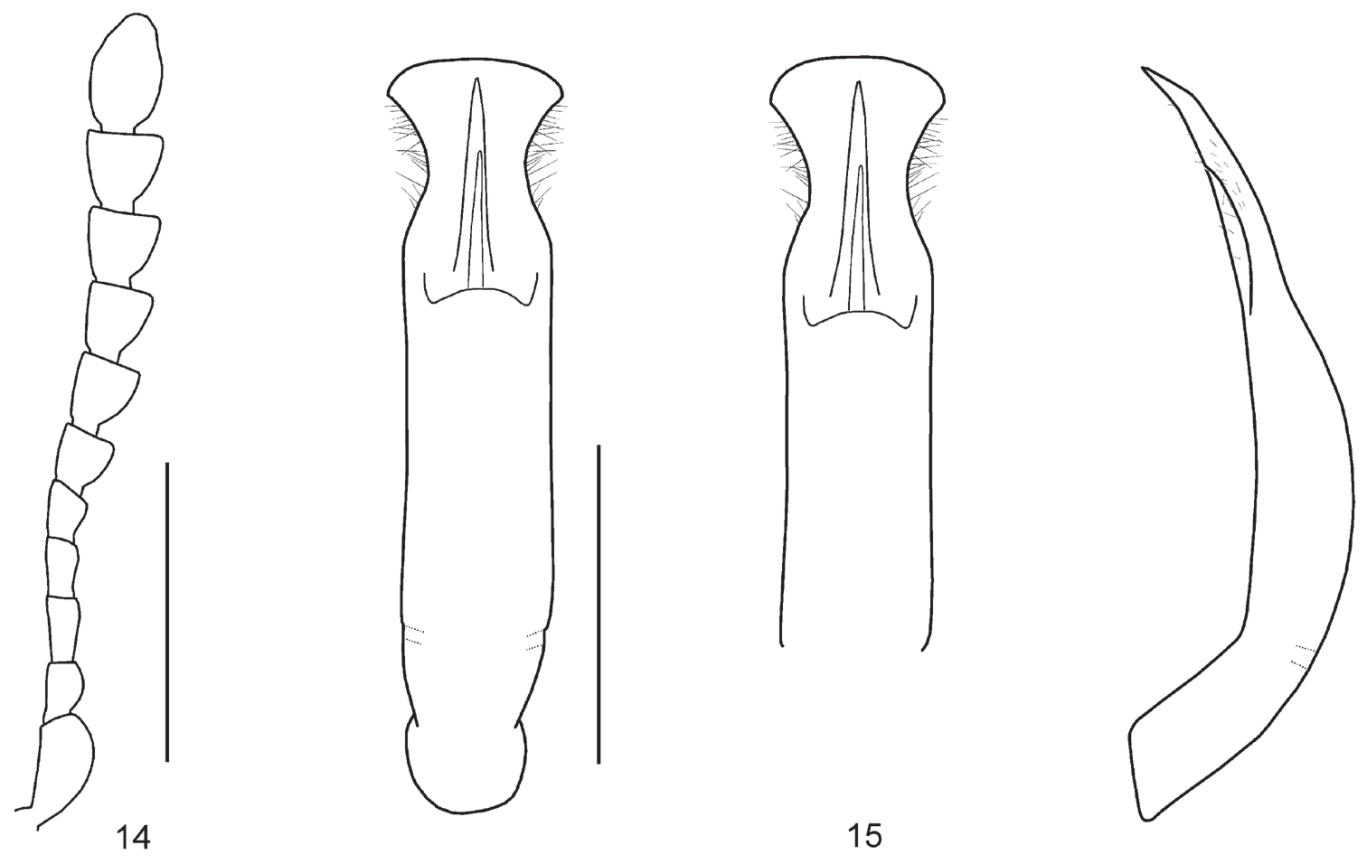
*Gonioctena mantillerii* sp. nov., holotype **14** antenna **15** aedeagus (dorsal view, apex in dorsal view, lateral view). Scale bars: 1.0 mm.

Figures 2 , 7 , 14 , 15

###### Type locality.

China: Sichuan.

###### Type material.

***Holotype*:** ♂ (MNHN), “Su-Tchuen, Chasseurs Indigènes 1903 // HOLOTYPUS Gonioctena (A.) mantillerii sp. n. Cho & Borowiec 2014”.

###### Diagnosis.

This new species is similar to *Gonioctena allardi* sp. nov. and *G. oudai* in body shape and coloration. However, *G. mantillerii* sp. nov. can be distinguished by the following characters: elytra covered with regular punctures arranged in single rows, partially irregular (rather irregular punctures arranged in confused single or double rows in *G. allardi* sp. nov., similar in *G. oudai*); pronotum without spots (same in *G. allardi* sp. nov., a pair of lateral obscure spots present in *G. oudai*); aedeagus with an apical process widened to lateral blunt projections, 1.2 × wider than the median lobe (widened to lateral tooth-like projections, 1.2 × wider than the median lobe in *G. allardi* sp. nov., very large, 1.5 × wider than the median lobe in *G. oudai*).

###### Description.

Measurements in mm (n = 1): length of body: 6.75; width of body: 4.85; height of body: 3.30; width of head: 2.00; interocular distance: 1.27; width of apex of pronotum: 2.35; width of base of pronotum: 4.42; length of pronotum along midline: 1.95; length of elytra along suture: 5.10.

Body short oval and strongly convex (Fig. 2). Head reddish brown, with apex of mandibles black. Antennomeres I–VI yellowish brown, VI partially darkened, VII–XI blackish brown. Pronotum reddish brown, basal margin black. Scutellum reddish brown. Elytra orange, with six pairs of black spots, dark area in median region. Venter and legs entirely reddish brown. ***Head*.** Vertex weakly convex, covered with sparse punctures, becoming coarser and denser toward sides. Frontal suture V-shaped, reaching anterior margin, coronal suture rather short. Frons flat, suddenly depressed at anterior margin, covered with dense punctures. Clypeus very narrow and trapezoidal. Anterior margin of labrum almost straight. Mandibles with two sharp apical teeth and deep excavation for apical maxillary palpomere on outer side. Maxillary palps four-segmented, with apical palpomere slightly widened, truncate apically. Antennae reaching pronotal base; antennomere I robust; antennomere II shorter than III; antennomere III longer than IV; antennomeres VII–X widened, VII–VIII slightly longer than wide, IX–X almost as long as wide; XI longest, ~ 1.65 × as long as wide (Fig. 14). ***Pronotum*.** Lateral sides widest at base, strongly and roundly narrowed anteriorly, anterior angles strongly produced (Fig. 7). Anterior and lateral margins bordered; lateral margins well visible in dorsal view. Trichobothria absent on both anterior and posterior angles. Disc covered with very sparse punctures; lateral sides covered with much larger and denser punctures; interspaces covered with fine and sparse punctures. Scutellum distinctly wider than long, narrowed posteriorly. ***Elytra*.** Lateral sides subparallel, widest near middle, thence roundly narrowed posteriorly. Humeral calli well developed. Disc covered with eleven regular rows of large punctures, including a short scutellar row, punctures partially irregular; interspaces covered with fine and sparse punctures. Epipleura visible except near base in lateral view. Hind wings well developed. ***Venter*.** Hypomera weakly rugose, with few punctures near anterolateral corners of prosternum. Prosternum covered with coarse and moderately dense punctures bearing long setae; prosternal process enlarged apically, bordered laterally, with sparse punctures. Metasternum covered with small and sparse punctures in median region, large and dense punctures in lateral region. Abdominal ventrites covered with sparse or moderately dense punctures bearing short setae. ***Legs*.** Moderately robust. Tibiae widened apically, with a tooth-like projection. Fore legs with tarsomere I slightly narrower than III. Tarsal claws appendiculate. ***Genitalia*.** Aedeagus rather thin, subparallel-sided, strongly concave in apical 1/4, setose apicolaterally, with the apical process widened to lateral blunt projections in dorsal view; moderately curved, apex pointed in lateral view (Fig. 15).

###### Etymology.

Dedicated to Antoine Mantilleri, the curator of beetles at the Museum National d’Histoire Naturelle in Paris.

###### Distribution.

China (Sichuan).

##### Gonioctena (Asiphytodecta) oudai

Taxon classificationAnimaliaColeopteraChrysomelidae

Cho & Borowiec, 2016

68A2E865-9963-507F-82B6-E0E6B191542E

Figures 3 , 8 , 16

Gonioctena (Asiphytodecta) oudai Cho & Borowiec, 2016b: 170 (type locality: China, Sichuan, Leshan, Mt. Emei).

###### Type material.

***Holotype*:** ♂ (NMPC), “China, SW Sichuan, Mt. Emei, 1000–2000 m, 6.VI.1997, Ouda M. lgt. // HOLOTYPUS Gonioctena (A.) oudai sp. n. Cho & Borowiec 2015”. ***Paratype***: 1♂ (LMC), “China: Sichuan, Mt. Emei, 600–1050 m, 5–19.V.1989, Lad. Bocak lgt. // PARATYPUS Gonioctena (A.) oudai sp. n. Cho & Borowiec 2015”.

###### Distribution.

China (Sichuan).

**Figures 16–18.**
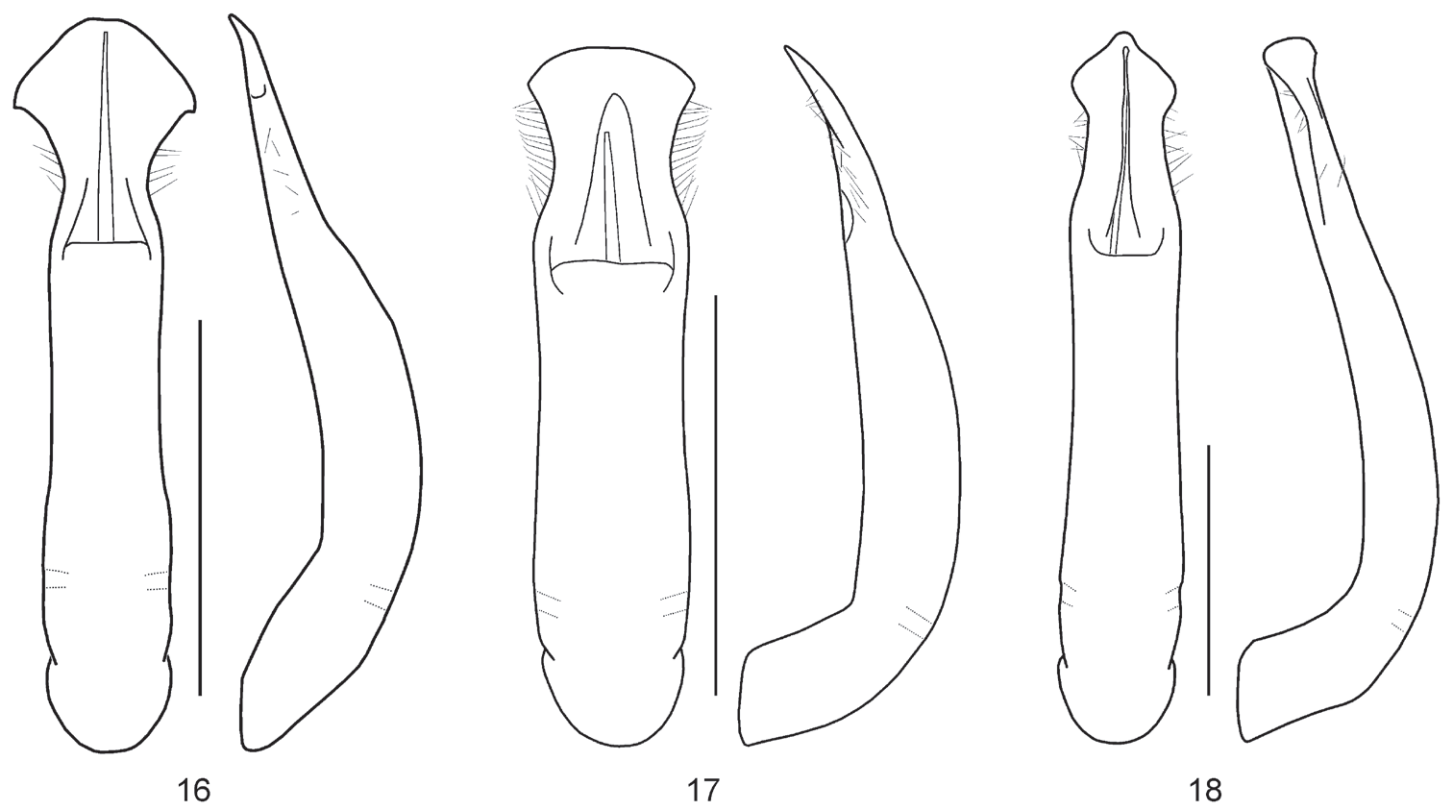
Aedeagus, dorsal, and lateral views (after Cho and Borowiec 2016b) **16***Gonioctena oudai***17***G. subgeminata***18***G. tonkinensis*. Scale bars: 1.0 mm.

##### Gonioctena (Asiphytodecta) subgeminata

Taxon classificationAnimaliaColeopteraChrysomelidae

(Chen, 1934)

8E92CE73-ADCE-5DF4-AEFB-6E8C00F53BE3

Figures 4 , 9 , 17

Phytodecta subgeminatus Chen, 1934: 71, 75 (type locality: China, Guangdong, Guangzhou), 1938: 290, 294.
Phytodecta (Asiphytodecta) subgeminatus : Chen 1935: 131, 1936: 88; Chûjô 1958: 67.
Asiphytodecta subgeminatus : Chen and Young 1941: 208.
Gonioctena (Asiphytodecta) subgeminata : Gressitt and Kimoto 1963: 365; Kimoto and Gressitt 1981: 385, 386; Kippenberg 2010: 432; Yang et al. 2014: 388; Yang et al. 2015: 54; Cho and Borowiec 2016b: 170, 174.
Gonioctena (Asiphytodecta) subgeminata subgeminata : Gressitt and Kimoto 1963: 360.
Gonioctena (Asiphytodecta) subgeminatus : Kimoto and Chu 1996: 52; Kimoto and Takizawa 1997: 158, 295, 369; Kimoto 2003: 79.
Gonioctena subgeminata : Yu et al. 1996: 68.

###### Type material.

Type probably lost.

###### Other material.

China – **Anhui**: 1♂ (TLMF), Tianzhushan env., 30.75N, 116.45SE, 11–14.V.2004, Jaroslav Turna leg.; **Fujian**: 1♂ (NHMB), Kuatun (2300 m), 27,40 n. Br. 117,40ö., 2.VI.1938, L.J. Klapperich leg.; 1♂ (BPBM), Shaowu, Taohulan, 21.II.1943, K.S. Liu leg.; **Guangdong**: 1♀ (BPBM), Yaoshan (Mt. range), Lin-hsien (District), 3.V.1934, F.K. To leg.; **Hunan**: 1♂ (NHMUK), mts. Dalongshan, XinHua, 1600 m, VII.2004, Jing leg.; 1♂ (NHMUK), mts. Wugongshan, ad An-Fu 1650 m, VIII.2004; **Jiangxi**: 2♀♀ (NHMUK), mts. Tianiangshan, ad Xin-Huan, 1600 m, VII.2004; **Sichuan**: 1♀ (NHMUK), Nanping, 6.VI.2001, E. Kučera leg.; 1♂ (HCC), Nanping-Jiuzhaigou, 7–12.VI.2009, E. Kučera leg.; 1♂, 1♀ (HCC), Nanping-Jiuzhaigou, 11–14.VI.2011, E. Kučera leg.; **Zhejiang**: 2♂♂ (NHMB), Tienmuschan; **Taiwan**: 1♀ (HTC), Guandaoxi, 5.V.1973, S. Nakamura leg.

###### Distribution.

China (Anhui, Fujian, Guangdong, Hunan, Jiangxi, Sichuan, Zhejiang), Taiwan.

###### Remarks.

Chen (1934) proposed this species based on two specimens from ZMHB with a reddish brown pronotum and elytra with eleven black spots including the obscure spot near the apex. Searching the ZMHB collection, I could not find any specimen matching the original description. However, the original description of *G. subgeminata*, with an illustration of the habitus, allowed its identification without any doubt.

##### Gonioctena (Asiphytodecta) tonkinensis

Taxon classificationAnimaliaColeopteraChrysomelidae

(Chen, 1934)

710AB8E9-EC28-5E72-B24D-18733F9CC0A2

Figures 5 , 10 , 18

Phytodecta subgeminatus var. tonkinensis Chen, 1934: 76 (type locality: Vietnam, Lang Son, Loc Binh, Mt. Mau Son), 1938: 295; Kimoto and Gressitt 1981: 386 (synonymized with G. subgeminata); Kippenberg 2010: 432 (as synonym of G. subgeminata).
Phytodecta (Asiphytodecta) subgeminatus var. tonkinensis : Chen 1935: 131, 1936: 88.
Asiphytodecta subgeminatus tonkinensis : Chen and Young 1941: 208.
Gonioctena (Asiphytodecta) subgeminata tonkinensis : Gressitt and Kimoto 1963: 360.
Gonioctena (Asiphytodecta) tonkinensis : Cho and Borowiec 2016b: 181 (resurrected as a valid species).

###### Type material.

***Lectotype*** (designated by Cho and Borowiec 2016b): ♀ (ZMHB), “Tonkin, Montes Mauson, April, Mai 2–3000’, H. Fruhstorfer // Type // TYPE // *Phytodecta subgeminata* Chen // LECTOTYPUS Phytodecta subgeminata var. tonkinensis Chen, 1934 des. H.W. Cho 2015”. ***Paralectotype***: 1♂ (ZMHB), same data as for lectotype.

###### Distribution.

China (Guangxi), Vietnam (Vinh Phuc).

###### Remarks.

*Gonioctena subgeminata tonkinensis* (Chen, 1934) was synonymized with *G. subgeminata* (Chen, 1934) by Kimoto and Gressitt (1981). However, this name was removed from synonymy with *G. subgeminata* and raised to the species level by Cho and Borowiec (2016b). Several larvae were dissected from a female specimen; therefore, this species is ovoviviparous.

## Supplementary Material

XML Treatment for Gonioctena (Asiphytodecta) allardi

XML Treatment for Gonioctena (Asiphytodecta) mantillerii

XML Treatment for Gonioctena (Asiphytodecta) oudai

XML Treatment for Gonioctena (Asiphytodecta) subgeminata

XML Treatment for Gonioctena (Asiphytodecta) tonkinensis
